# The Voting Paper and the Prizes

**Published:** 1914-05-09

**Authors:** 


### The Voting Paper and the Prizes.

We give below the voting paper which should be filled in by all desiring to compete in the voting for the best ward locker, with directions for voting. **To the competitors whose designs are placed first and second, a prize of Five Guineas and Two Guineas respectively will be awarded. To the voter whose coupon is found most nearly to agree with the majority of opinions expressed by the votes a special prize of Three Guineas will be awarded.**

"The Hospital," May 9.

VOTING COUPON.

Institution Efficiency and Economy Prize Scheme.

WARD LOCKER.

